# Made In Germany – traumatische Myiasis im (Klima‑)Wandel

**DOI:** 10.1007/s00113-025-01643-1

**Published:** 2025-10-20

**Authors:** Anna-Lena Hauser, Christiane Kruppa, Thomas Armin Schildhauer

**Affiliations:** 1 Orthopädische Universitätsklinik am St. Josef Hospital, Gudrunstraße 56, 44791 Bochum, Deutschland; 2 BG Universitätsklinikum Bergmannsheil Bochum, Bochum, Deutschland

**Keywords:** Komplikationsmanagement, Klimawandel, Wundinfektion, Trauma, Maden, Complication management, Maggots, Trauma, Climate change, Wound infestation

## Abstract

**Hintergrund:**

In tropischen Regionen ist die traumatische Myiasis seit Langem als chirurgische Komplikation bekannt. Als Folge des Klimawandels migrieren die pathogenen Fliegen in nördlichere Klimazonen, sodass das Krankheitsbild auch in Europa prävalenter wird.

**Fragestellung:**

Es gibt aktuell keine Daten aus Deutschland über die patientenbezogenen Risikofaktoren, die Häufigkeit, den Verlauf und die zu erwartenden Komplikationen. Diese Fallserie soll diese Lücke schließen. Ebenso soll ein Zusammenhang mit Wetterdaten diskutiert werden.

**Material und Methoden:**

In dieser retrospektiven Fallanalyse von 6 Patienten werden Risikofaktoren, systemisch-septische Komplikationen, Behandlungsschemata, die Länge der Krankenhausaufenthalte und Wetterdaten diskutiert.

**Ergebnisse:**

Patienten waren im Median 60,5 Jahre alt und hatten 4 Vorerkrankungen. Risikofaktoren waren männliches Geschlecht (67 %), sozioökonomische Benachteiligung (84 %), schlechte Körperhygiene (67 %), arterieller Hypertonus (84 %), Alkoholismus (50 %) und Verletzungen der unteren Extremität (100 %, 67 % frakturassoziiert). Die mittlere Krankenhausaufenthaltsdauer betrug 49 ± 25,39 Tage. In allen Fällen wurde eine bakterielle Superinfektion nachgewiesen.  Alle stationär aufgenommenen Fälle wurden operativ, 80 % begleitend antibiotisch behandelt. In 33 % der Fälle war eine Amputation notwendig. Kein Patient musste aufgrund einer Septikämie intensivmedizinisch behandelt werden. Alle Fälle traten im Sommer und im Spätfrühling auf. Am Aufnahmetag betrug die Temperatur tagsüber im Median 20,6 °C und nachts 14,6 °C bei einer medianen Luftfeuchtigkeit von 66,6 %; die Temperaturen tagsüber lagen am Aufnahmetag höher als in der Woche zuvor und im Monat zuvor. Bezüglich der Luftfeuchtigkeit gab es ebenso keine statistisch signifikanten Unterschiede zwischen den 3 Vergleichszeitpunkten.

**Diskussion:**

Die traumatische Myiasis ist in Europa angekommen. Temperatur- und ggf. auch Feuchtigkeitsspitzen könnten das Schlüpfen der Maden triggern und damit die klinische Präsentation begünstigen.

## Abstract (en)

**Background:**

In tropical regions traumatic myiasis has long been recognized as a surgical complication. As a result of climate change pathogenic flies are migrating to more northern climates making this condition increasingly more prevalent in Europe.

**Objective:**

Currently, no data from Germany exist regarding the patient-related risk factors, incidence, clinical course and expected complications. This case series aims to fill that gap. Additionally, a relationship with weather data is discussed.

**Material and methods:**

This retrospective case analysis of six patients discusses the risk factors, systemic septic complications, treatment protocols, length of hospital stay and weather data.

**Results:**

The median patient age was 60.5 years with an average of 4 pre-existing conditions. Risk factors included male gender (67%), socioeconomic disadvantage (84%), poor personal hygiene (67%), arterial hypertension (84%), alcoholism (50%) and lower extremity injuries (100%, 67% fracture-related). The mean hospital stay was 49 ± 25.39 days. All cases showed evidence of a bacterial superinfection. Of the admitted cases 100% underwent surgery and 80% additionally received antibiotic treatment. Amputation was required in 33% of cases. No patients required intensive care treatment for septicemia. All cases occurred in late spring and summer. On the day of admission the median daytime temperature was 20.6 °C and 14.6 °C at night with a median humidity of 66.6%. Daytime temperatures were higher on the day of admission than in the preceding week and month. There were no statistically significant differences in humidity between the three time points.

**Conclusion:**

Traumatic myiasis has arrived in Europe. Temperature spikes and possibly humidity peaks, could trigger larval hatching and thereby favor the clinical presentation.

Im Kontext des Klimawandels verändern sich die Krankheitsbilder und Erregerspektren der septischen Chirurgie. Die veränderten klimatischen Bedingungen in Deutschland, die Einschleppung fremder Fliegenarten durch Migration, z. B. durch Klimaflüchtlinge oder Reisende, sowie die gesteigerte Vulnerabilität unserer beständig älter werdenden Patienten erhöhen die Prävalenz der traumatischen Myiasis, also des Befalls von Wunden durch Fliegenmaden [3, 10, 20, 29].

## Pathophysiologie und Abhängigkeit vom Klima

Der alkalische Geruch von Wunden lockt *Diptera* an [13], die nach etwa 6 Tagen Larven in ebenjene Wunden legen [6], aus denen schließlich Maden schlüpfen. Dies führt zum einen zu einer Vergrößerung der Wundoberfläche sowie zu einer Vertiefung der Wunden [25, 27], zum anderen zu bakteriellen Superinfektionen insbesondere mit *Staphylococcus aureus, Pseudomonas aeruginosa, Stenotrophomonas maltophilia* und *Ignatzschineria indica* [1, 22].

Patientenbezogene Risikofaktoren sind vielfältig. Traumatische Myiasis wird mit schlechter Zahnhygiene, Malignomen, schädlichem Alkoholgebrauch, supportiven Läsionen, schlechter Körperhygiene und niedrigem sozioökonomischen Status in Verbindung gebracht [10]. Auch Hautkrankheiten begünstigen die Kolonisierung [26].

Im Allgemeinen ist die Prävalenz in tropischen und subtropischen Regionen deutlich höher, da das dort vorherrschende warme, feuchte Klima optimale Reproduktionsbedingungen für die Insekten bietet [10, 13]. Der Klimawandel schafft auch in Europa neue Lebensräume und ökologische Nischen für tropische Insekten [11]. Berichte über einen Madenbefall von Wunden nehmen tendenziell in den Sommermonaten zu [26].

## Material und Methoden

In dieser explorativ-retrospektiven Fallserie wurden Patienten mit traumatischer Myiasis, die in unserer Universitätsklinik mit überregionalem Traumazentrum behandelt wurden, identifiziert.

Zunächst wurde eine Patientenabfrage unter Verwendung der ICD-10-Codes (International Statistical Classification of Diseases and Related Health Problems, 10. Revision) M60.0- aller Patienten durchgeführt, die im Zeitraum Oktober 2012 bis Oktober 2022 behandelt wurden. Anschließend wurde eine Datenanalyse anhand von Krankenakten, Ambulanzakten und Operationsberichten aus dem Archiv und von elektronischen Arbeitsplätzen durchgeführt.

In die Auswertung wurden Patienten einbezogen, bei denen im Zeitraum Oktober 2012 bis Oktober 2022 eine traumatische Myiasis korrekt diagnostiziert wurde und deren Behandlung in unserer Einrichtung stattfand. Die Diagnosekriterien waren wie folgt: Vorhandensein einer Wunde oder eines Hautdefekts, sichtbarer Nachweis von Maden, lokale oder fortgeleitete Entzündungszeichen (Rötung, lokale Schmerzhaftigkeit, Überwärmung, Schwellung, systemische Infektionszeichen). Sie wurden ausgeschlossen, wenn diese Kriterien nicht erfüllt waren, und wenn die Diagnose post factum als falsch identifiziert wurde.

Primäre Studienziele waren der Zusammenhang zwischen Klima- und Wetterdaten und dem Auftreten von traumatischer Myiasis in den letzten 10 Jahren und die Bestimmung patientenbezogener Risikofaktoren für Insektenbefall. Für Letzteres wurden das Vorhandensein von Vorerkrankungen wie beispielsweise Diabetes mellitus, Niereninsuffizienz, Leberfunktionsstörungen ebenso wie die Einnahme immunsupprimierender Medikamente untersucht. Des Weiteren wurden die Wundanamnese (Trauma, postoperative Wundheilungskomplikation, Gefäßläsion) sowie die Wundlokalisation erfragt. Therapiespezifische Variablen (Art der operativen Behandlung, Verwendung eines vakuumversiegelten Verbandes (VAC), antibiotische Behandlung, Erzielen des Wundverschlusses und damit verbundene Komplikationen wie z. B. Wundheilungsstörungen, Infektionen, Notwendigkeit einer Revisionsoperation, Veränderungen des Keimspektrums, Notwendigkeit einer intensivmedizinischen Behandlung, Sepsis, Tod, Dauer des Krankenhaus- und Intensivaufenthalts) wurden ebenso untersucht wie infektionsspezifische Faktoren wie Keimspektrum und Ausmaß der Entzündung, für die die Körpertemperatur sowie die Konzentration des C‑reaktiven Proteins (CRP) im Plasma des Patienten und die Leukozytenzahl als Proxy-Variablen verwendet wurden.

Sekundäre Ergebnisvariablen waren demografische Daten (Alter, Geschlecht), Sozialanamnese, Body Mass Index (BMI), Pflegestatus, Mortalität und Komplikationen während des Krankenhausaufenthalts.

Alle Wetterdaten wurden von https://www.timeanddate.de/wetter/deutschland/bochum/rueckblick?month=8&year=2020 gesammelt.

Die mittlere Luftfeuchtigkeit sowie die Tages- und Nachttemperaturen am Aufnahmetag wurden als Mittelwert von 4 Temperatur- oder Luftfeuchtigkeitsmessungen innerhalb eines Zeitraums von 12 h berechnet. Die gleichen Parameter wurden für die Woche und den Monat vor der Aufnahme mit der gleichen Methode berechnet. Ein Monat wurde als 30 Tage definiert. Die statistische Auswertung beinhaltete zunächst einen Shapiro-Wilk-Test auf Normalverteilung und dann eine Analyse mittels ANOVA.

## Ergebnisse

Wir schlossen 6 Patienten ein, von denen 5 männlich und einer weiblich war. Das Medianalter betrug 60,5 Jahre (Spanne: 27 bis 83 Jahre), der mediane BMI 27,2 kg/m^2^ (Spanne: 24,69–31,18 kg/m^2^) und der mediane ASA-Score 3 (Spanne: 2–4).

Die eingeschlossenen Patienten wiesen im Median 4 Vorerkrankungen auf (Spanne: 2–8). 5 von 6 Patienten litten an arterieller Hypertonie, 3/6 Patienten gaben schädlichen Alkoholkonsum an. 2/6 Patienten litten an einer peripheren arteriellen Verschlusskrankheit und Diabetes mellitus *Typ 2*. Ein Patient wies eine orale Immunsuppression auf (Tab. 1).

**Tab. 1:** Vorerkrankungen und Sozialanamnese

Patientennummer	Vorerkrankungen	Sozialanamnese
1	Arterieller Hypertonus, Nikotin- und schädlicher Alkoholgebrauch, Asthma bronchiale	Lebt allein, keine Pflegestufe, schwer reduzierte Körperhygiene
2	Arterieller Hypertonus, Epilepsie, Mitralklappen- und Trikuspidalklappeninsuffizienz, AV-Block 1. Grades, rheumatoide Arthritis, Demenz, Schwindel unklarer Ursache	Kurzzeitpflege, schwer reduzierte Körperhygiene
3	Arterieller Hypertonus, paranoide Schizophrenie, schädlicher Alkoholgebrauch	Pflegeheim, schwer reduzierte Körperhygiene
4	Arterieller Hypertonus, periphere arterielle Verschlusskrankheit, Diabetes mellitus Typ 2	Unauffällig
5	Periphere arterielle Verschlusskrankheit, Diabetes mellitus Typ 2	Unauffällig
6	Drogengebrauch, schädlicher Alkoholgebrauch	Wohnungslos, schwer reduzierte Körperhygiene

Alle Wunden befanden sich an den unteren Extremitäten. 4/6 Patienten wiesen Wunden am Knöchel auf, 3 davon nach Knöchelfrakturen (Tab. 2). Allen Patienten wurde die Aufnahme angeboten. 1/6 entschied sich gegen ärztlichen Rat für die Entlassung. Die Dauer des Krankenhausaufenthalts der übrigen Patienten betrug im Median 49 Tage (Range: 2 bis 63 Tage).

**Tab. 2:** Primäre Outcome-Variablen

	Lokalisation der Wunde	Wundanamnese	Isolierte Erreger	Operative Behandlung	Antibiotische Behandlung
1	Knöchel, links	Septisches Empyem bei Zustand nach Knöchelarthroskopie mit multiplen Revisionsoperationen; zuletzt Anlage eines Ilizarov-Fixateurs	*Klebsiella pneumoniae*	2 operative Débridements, VAC-Therapie, Spalthautdeckung	Ja
2	Knöchel, rechts	Trimalleoläre Fraktur mit Ilizarov-Fixateur-Therapie nach verfrühter Belastung	*Staphylococcus aureus*, *Providencia rettgeri, Oligella ureolytica*	Metallentfernung, Anlage eines AO-Fixateurs, VAC-Therapie, Spalthautdeckung	Ja
3	Knöchel, links	Sprunggelenkluxationsfraktur mit postoperativer Wundheilungsstörung	*Proteus mirabilis*	Metallentfernung, Débridement, offene Wundbehandlung	Nein
4	Knöchel, links	Überrolltrauma, Sprunggelenkluxationsfraktur mit Behandlung im AO-Fixateur	*Aeromonas hydrophila, Enterobacter cloacae, Enterococcus faecalis, Staphylococcus epidermidis, Klebsiella*-Spezies, *Candida*-Spezies, *Cutibacterium avidum, Stenotrophomonas maltophilia*	Wundrevision (3-mal), Coldex-Therapie, Resektion der Hallux -longus-Sehne, anterolateraler Oberschenkel(ALT)-Lappen, Anastomosenresektion, Lappenresektion, Débridement + VAC-Therapie, Resektionsarthroplastie des linken Knöchels, Nekrosektomie, Unterschenkelamputation	Ja
5	Digitus II, linker Fuß	Periphere arterielle Verschlusskrankheit	*Proteus mirabilis, Enterococcus faecalis*	TMT-Amputation	Ja
6	Unterschenkel, rechts	Unklar	Keine Testung	Entlassung gegen ärztlichen Rat	

*VAC-Therapie* Vacuum assisted closure/Negative-pressure wound therapy, *AO* Arbeitsgemeinschaft für Osteosynthesefragen, *ALT-Lappen* Anterolateral thigh flap/anterolateraler Oberschenkellappen, *TMT *Tarsometatarsalgelenk, *+* und

In allen Wundabstrichen der stationär aufgenommenen Patienten konnten Bakterien nachgewiesen werden. Von einer reiner Besiedelung ist bei klinischer Infektionssymptomatik nicht auszugehen. Insgesamt wurden 12 verschiedene pathogene bzw. fakultativ pathogene Erreger isoliert, von denen einer fungal und die übrigen bakteriell waren. Von Letzteren waren 7 gramnegativ, 4 grampositiv und 8 fakultativ oder ausschließlich anaerob. Alle Patienten wurden operativ behandelt und in 4 von 5 Fälle durch eine Antibiotikabehandlung unterstützt. In 2 der 5 Fälle war eine Amputation erforderlich. 3 von 5 Patienten erhielten eine Wundkonditionierung mittels Vakuumverbänden (Tab. 2).

Kein Patient wurde wegen einer wundbedingten Septikämie auf die Intensivstation verlegt. Die höchste im stationären Aufenthalt laborchemisch gemessene Konzentration des C‑reaktiven Proteins betrug im Median 6,8 mg/dl (Spanne: 5,6–36,1 mg/dl), die höchste im stationären Aufenthalt laborchemisch gemessene Leukozytenzahl im Median 12,7/nl (Spanne: 6,1–24,9/nl) und die höchste dokumentierte Körpertemperatur im Median 37,6 °C (Spanne: 36,9–38 °C).

4 Fälle traten im Sommer auf, 2 im späten Frühjahr.

**Abb. 1:**
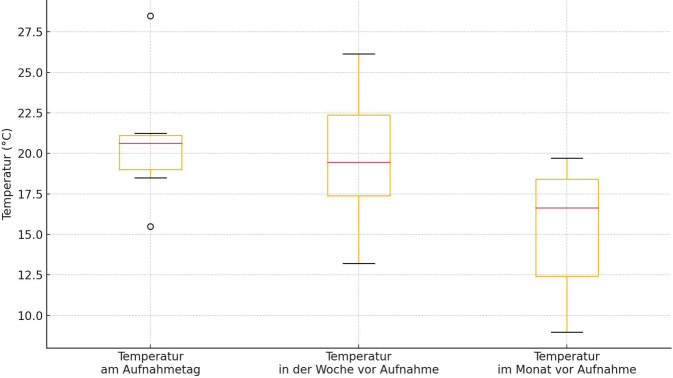
Temperaturen tagsüber am Aufnahmetag, in der Woche zuvor und im Monat zuvor

Am Tag der Aufnahme betrug die Temperatur im Median tagsüber 20,6 °C (Spanne: 15,5–28,5 °C) und nachts 14,6 °C (Spanne: 10,25–21,25 °C) bei einer medianen Luftfeuchtigkeit von 66,6 % (Spanne: 55,25–78,5 %). Die Temperatur am Aufnahmetag war dabei tagsüber sowohl höher als in der Woche zuvor (Abb. 1; 19,4 °C, Spanne 13,21–26,14 °C, *p* = 0,48) als auch im Monat zuvor (tagsüber 16,65 °C, Spanne 8,97–19,71, *p* = 0,056).

**Abb. 2:**
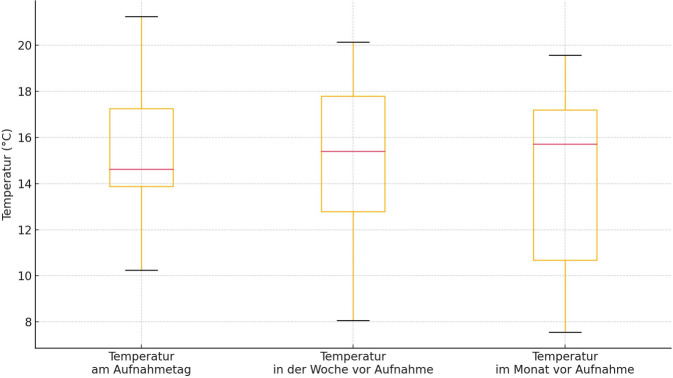
Temperaturen nachts am Aufnahmetag, in der Woche zuvor und im Monat zuvor

Nachts lag sie tiefer als in der Woche (Abb. 2; 15,40 °C, Spanne 8,07–20,14, *p* = 0,84) und im Monat zuvor (15,71 °C, Spanne 7,55–19,56 °C, *p* = 0,64).

**Abb. 3:**
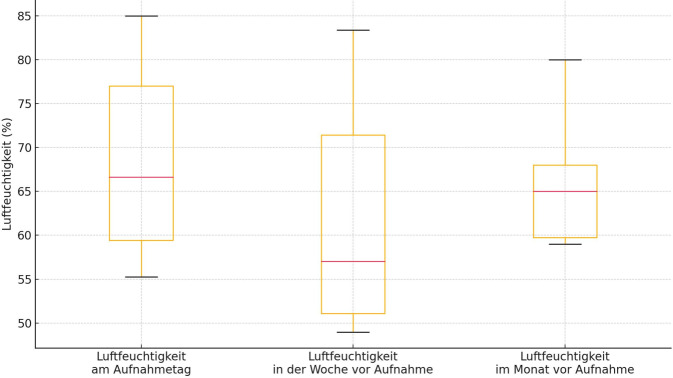
Luftfeuchtigkeit am Aufnahmetag, in der Woche zuvor und im Monat zuvor

Bezüglich der Luftfeuchtigkeit gab es ebenso keine statistisch signifikanten Unterschiede zwischen den 3 Vergleichszeitpunkten (Tag der Aufnahme, Median in der Woche vor Aufnahme, Median im Monat vor Aufnahme). Die Luftfeuchtigkeit betrug im Median 66,62 % (59–85 %) am Aufnahmetag, 57,04 % in der Woche vor Aufnahme (49–83 %), sowie 65 % im Monat vor der stationären Aufnahme (59–80 %) (Abb. 3).

## Diskussion

Die Bedrohung der menschlichen Zivilisation durch den Klimawandel wurde bereits durch eine Vielzahl im Ergebnis ungemein kohärenter Studien belegt [9, 19]. In wärmeren Regionen endemische Insekten werden nun zunehmend auch in Europa heimisch [7, 18, 28]. Krankheiten wie beispielsweise die traumatische Myiasis, die in subtropischen und tropischen Breitengraden schon seit jeher bekannt sind, verbreiten sich nun auch in nördlichen Gefilden [9, 12, 28]. Obwohl sich der Hygienestandard hier in den vergangenen Jahrzehnten stark verbesserte [2, 14], ist dieser aber für diverse Untergruppen der Bevölkerung wie sozioökonomisch Benachteiligte und Obdachlose nach wie vor schlecht [4, 5, 7].

Unsere, wenngleich kleine, Fallserie inkludiert die größte Anzahl an Patienten mit Wundmyiasis in Mitteleuropa in der aktuellen Literatur, in der bislang nur vereinzelte Fallberichte aus Europa existieren, und lässt uns einige wichtige Schlussfolgerungen ziehen.

Die Wundmyiasis ist, insbesondere angesichts des Klimawandels, eine klinisch relevante Pathologie.

Ihr Auftreten nimmt mit wärmerem Wetter zu, was in den kommenden Jahren und Jahrzehnten immer häufiger der Fall sein wird. Alle von uns erhobenen Fälle traten in den warmen Frühlings- und Sommermonaten auf. Die Durchschnittstemperaturen am Aufnahmetag sowie in der Woche zuvor lagen tagsüber tendenziell über dem Monatsdurchschnitt. Dies stimmt mit wissenschaftlichen Erkenntnissen, die eine erhöhte Verpuppungsgeschwindigkeit bei höheren Temperaturen belegen, überein [16], sowie mit anderen Fallberichten aus Europa und Nordamerika, die ein fast ausschließliches Auftreten der Wundmyiasis im Sommer [17, 21, 26] und eine Ausbreitung nach Norden infolge des Klimawandels beobachteten.

Die Wundmyiasis ist keine „demokratische“ Krankheit.

Die Patienten unseres Studienkollektivs weisen überproportional häufig mangelnde Körperhygiene auf (4/6 Fällen) oder leben nicht selbstständig in der eigenen Wohnung (3/6 Fällen). Sie haben in der Regel eine umfangreiche Krankengeschichte, insbesondere oberflächliche oder tiefe Hautgeschwüre oder Frakturen, die häufig mit externen Fixateuren behandelt werden. Dies stimmt mit früher berichteten Kollektiven überein [24, 30]. Sherman analysierte ein Kollektiv von 42 Fällen von Wundmyiasis in einer multizentrischen Studie in den USA. 75 % der Befallsfälle traten an Wunden der unteren Extremitäten auf [26], was mit den Wundlokalisationen in unserem Kollektiv übereinstimmt, und mindestens ein Drittel war obdachlos. Diese Quote war in unserem Kollektiv niedriger, was wohl auf den deutschen Sozialstaat zurückzuführen ist, obwohl auch in unserem Kollektiv häufig schwere Beeinträchtigungen der Körperhygiene auftraten. Obwohl die Patienten in unserem Zentrum tendenziell jünger waren, war die medizinische Vorgeschichte einschließlich der Häufigkeit von peripheren Gefäßerkrankungen, Alkoholismus und Diabetes mellitus ähnlich [26].

*Wir müssen (be-)handeln – aber wie?*

Auch in Ermangelung einer adäquaten Datenmenge gibt es derzeit keinen Konsens darüber, ob ein chirurgisches Wund-Débridement im Vergleich zu einer konservativen Behandlung zu einem positiven Outcome im Sinne einer schnelleren Wundkonsolidierung führt. Natürlich gelten die allgemeinen Grundsätze für komplizierte Wunden auch bei Myiasis. Die Behandlung besteht aus drei Säulen – radikales Débridement, lokale Anwendung von Antiseptika und antibiotische Therapie [8]. Die antibiotische Behandlung wird oft dadurch erschwert, dass die Bakterien häufig Resistenzen gegen mindestens eine antibiotische Substanz aufweisen [7]. Die antibiotische Behandlung sollte auf jeden Fall gramnegative Bakterien umfassen, da diese in madenbefallenen Wunden besonders häufig vorkommen. Diese Empfehlung deckt sich mit unseren Daten, in denen alle Wunden mindestens ein gramnegatives Bakterium aufwiesen, und der aktuellen Literatur [7], in der die These postuliert wird, dass Fliegenlarven das Wachstum von grampositiven Bakterien hemmen. Ein radikales Débridement ist unvermeidlich, da Maden entfernt werden müssen, da (a), wie beschrieben, Maden Bakterien übertragen und (b) infizierte Wunden Fliegen, die die Wunde vergrößern und vertiefen, weiteranziehen [23]. McIntosh et al. berichteten über die Wirksamkeit der lokalen Anwendung alkoholischer Lösungen in Bezug auf die Larvenmortalität, wobei allerdings die Gewebetoxizität und die Auswirkungen auf die Wundheilung zu berücksichtigen sind [23]. Wunden können entweder durch NPWT (Unterdruck-Wundtherapie) oder durch Wundauflagen behandelt werden. In unserem Zentrum wird beides durchgeführt. Die NPWT-Therapie zeigt günstige Ergebnisse hinsichtlich der Bildung von Granulationsgewebe, der Verringerung der Wundoberfläche und der Heilungszeit [15]. In Zukunft werden Studien erforderlich sein, um festzustellen, welche Mittel eine schnelle Wundheilung bei madenbefallenen Wunden wirksam fördern können, da es in der aktuellen Literatur an evidenzbasierten Empfehlungen fehlt.

Präventive Maßnahmen werden immer wichtiger und können so einfach sein wie der Einbau von Fliegengittern, die Reduktion der für den Verbandwechsel nötigen Zeit und die Klimatisation von Krankenhäusern trotz steigender Energiekosten [21]. Ebenso müssen wir die Terminierung elektiver Operationen an das Wetter anpassen und vulnerable Bevölkerungsgruppen angemessen schützen. Insbesondere Pflegeheime müssen das Risiko ihrer besonders gefährdeten Bewohner, an Myiasis zu erkranken, durch adäquate Screening- und Präventionsmaßnahmen senken. Primärversorger müssen darin geschult werden, Fliegenlarven frühzeitig zu erkennen. Die Wundversorgung muss auch und gerade für sozial ausgegrenzte Bevölkerungsgruppen zugänglich sein.

## Fazit für die Praxis

- Die traumatische Myiasis wird in Europa zunehmend häufiger werden.
- Die chirurgische Wundversorgung sollte ein Débridement enthalten, um eine sekundäre Wundvergrößerung zu vermeiden und das Risiko einer Superinfektion zu senken. Als Ultima Ratio ist eine Amputation zu erwägen.
- Ob lokale Wundtherapien Vorteile haben, ist umstritten.
- Madenbefallene Wunden sind häufig superinfiziert. Diese Bakterien weisen meistens mindestens eine Resistenz auf; häufig sind sie multiresistent.
- Eine antibiotische Therapie sollte erfolgen und kalkuliert gramnegative Erreger erfassen, bei Vorliegen der mikrobiologischen Ergebnisse ist ggf. eine Anpassung der antibiotischen Therapie erforderlich.
- Präventivmaßnahmen sind quintessenziell und können Fliegengitter, Klimatisation von Krankenhäusern, Reduktion von Wundexposition und Anpassung von Operationszeitpunkten an das erwartete Wetter beinhalten.

## Abkürzungen

ALT-Lappen: „Anterolateral thigh (flap)“ (anterolaterale Oberschenkel[lappen])
AO: Arbeitsgemeinschaft für Osteosynthesefragen
ASA: American Society of Anesthesiologists
AV-Block: Atrioventrikulärer Block
BMI: Body-Mass-Index
CRP: C‑reaktives Protein
ICD-10: International Statistical Classification of Diseases and Related Health Problems, 10. Revision
TMT: Tarsometatarsalgelenk
VAC: Vacuum Assisted Closure (vakuumversiegelter Verband)

## References

[1] Africano FJ, Faccini-Martinez AA, Pérez CE et al (2015) Pin-site myiasis caused by screwworm fly, Colombia. Emerging Infect Dis 21:905 10.3201/eid2105.141680 PMC4412229 25898067

[2] Aiello AE, Larson EL, Sedlak R (2008) Hidden heroes of the health revolution sanitation and personal hygiene. Am J Infect Control 36:S128–S151 19081496 10.1016/j.ajic.2008.09.008

[3] Alizadeh M, Mowlavi G, Kargar F et al (2014) A review of myiasis in Iran and a new nosocomial case from Tehran, Iran. J Arthropod Borne Dis 8:124 26114125 PMC4478423

[4] Amendt J (2013) Insektenbefall lebender Menschen–Zeichen der Vernachlässigung. Klinisch-forensische Medizin: Interdisziplinärer Praxisleitfaden für Ärzte, Pflegekräfte. In: Juristen und Betreuer von Gewaltopfern, S 493–497

[5] Anthonj C, Poague KIHM, Fleming L et al (2024) Invisible struggles: WASH insecurity and implications of extreme weather among urban homeless in high-income countries—A systematic scoping review. Int J Hyg Environ Health 255:114285 37925888 10.1016/j.ijheh.2023.114285

[6] Bandara W, Karunaratne W, Fuward R et al (2022) Estimating colonization time of maggots infesting wounds in dogs: three case studies. Can Soc Forensic Sci J 55:171–180

[7] Bernhardt V, Finkelmeier F, Tal A et al (2018) Multispecies blow fly myiasis combined with hypothermia in a man assumed to be dead. Parasitol Res 117:579–583 29170873 10.1007/s00436-017-5691-8

[8] Bischoff M, Kinzl L, Schmelz A (1999) Die komplizierte wunde. Unfallchirurg 102:797–804 10525624 10.1007/s001130050483

[9] Chitre SD, Crews CM, Tessema MT et al (2024) The impact of anthropogenic climate change on pediatric viral diseases. Pediatr Res 95:496–507 38057578 10.1038/s41390-023-02929-z PMC10872406

[10] Chodankar N, Dhupar V, Akkara F et al (2022) Oral and Maxillofacial Myiasis: A Literature Review. Medico Res Chronicles 9:121–132

[11] Dayrit JF, Sugiharto A, Coates SJ et al (2022) Climate change, human migration, and skin disease: is there a link? Int J Dermatol 61:127–138 33971021 10.1111/ijd.15543 PMC9158751

[12] El-Sayed A, Kamel M (2020) Climatic changes and their role in emergence and re-emergence of diseases. Environ Sci Pollut Res Int 27:22336–22352 32347486 10.1007/s11356-020-08896-w PMC7187803

[13] Francesconi F, Lupi O (2012) Myiasis. Clin Microbiol Rev 25:79–105 22232372 10.1128/CMR.00010-11 PMC3255963

[14] Gates S, Hegre H, Nygård HM et al (2012) Development consequences of armed conflict. World Dev 40:1713–1722

[15] Gottipati S, Gowtham B, Chalimeda S et al (2024) Functional Outcomes in Orthopaedic Open Wounds Treated With Vacuum-Assisted Closure Therapy: A Prospective Case Series. Cureus 16:10.7759/cureus.54468 PMC10953839 38510913

[16] Grassberger M, Reiter C (2002) Effect of temperature on development of Liopygia (= Sarcophaga) argyrostoma (Robineau-Desvoidy)(Diptera: Sarcophagidae) and its forensic implications. J Forensic Sci 47:1332–1336 12455659

[17] Greenberg B (1984) Two cases of human myiasis caused by Phaenicia sericata (Diptera: Calliphoridae) in Chicago area hospitals. J Med Entomol 21:615–615 6502618 10.1093/jmedent/21.5.615

[18] Hawkes WL, Walliker E, Gao B et al (2022) Huge spring migrations of insects from the Middle East to Europe: quantifying the migratory assemblage and ecosystem services. Ecography 2022:e6288

[19] Hulme M, Barrow EM, Arnell NW et al (1999) Relative impacts of human-induced climate change and natural climate variability. Nature 397:688–691

[20] Lageju N, Neupane D, Jaiswal LS et al (2022) Pin-tract myiasis after external bone fixation: A case report and review of literature. Int J Surg Case Rep 95:107247 35636216 10.1016/j.ijscr.2022.107247 PMC9149183

[21] Lukin LG (1989) Human cutaneous myiasis in Brisbane: a prospective study. Med J Aust 150:237–240 2716619 10.5694/j.1326-5377.1989.tb136454.x

[22] Sayhood MH, MMFaa ALMM (2022) Classical and molecular identification of Staphylococcus aureus isolated from infestation cattle wounds with myiasis in Basrah governorate, Iraq. IJVS 36:641–646

[23] Mcintosh MD, Merritt RW, Kolar RE et al (2011) Effectiveness of wound cleansing treatments on maggot (Diptera, Calliphoridae) mortality. Forensic Sci Int 210:12–15 21377818 10.1016/j.forsciint.2011.01.028

[24] Park P, Lodhia K, Eden S et al (2005) Pin-site myiasis: a rare complication of halo orthosis. Spinal Cord 43:684–686 15968303 10.1038/sj.sc.3101773

[25] Robbins K, Khachemoune A (2010) Cutaneous myiasis: a review of the common types of myiasis. Int J Dermatol 49:1092–1098 20883399 10.1111/j.1365-4632.2010.04577.x

[26] Sherman RA (2000) Wound myiasis in urban and suburban United States. Arch Intern Med 160:2004–2014 10888974 10.1001/archinte.160.13.2004

[27] Singh A, Singh Z (2015) Incidence of myiasis among humans—a review. Parasitol Res 114:3183–3199 26220558 10.1007/s00436-015-4620-y

[28] Trájer AJ (2021) Aedes aegypti in the Mediterranean container ports at the time of climate change: A time bomb on the mosquito vector map of Europe. Heliyon 7:e7981 34568601 10.1016/j.heliyon.2021.e07981 PMC8449062

[29] Uysal S, Ozturk AM, Tasbakan M et al (2018) Human myiasis in patients with diabetic foot: 18 cases. Ann Saudi Med 38:208–213 29848939 10.5144/0256-4947.2018.208 PMC6074300

[30] Verettas DA, Chatzipapas CN, Drosos GI et al (2008) Maggot infestation (myiasis) of external fixation pin sites in diabetic patients. Trans R Soc Trop Med Hyg 102:950–952 18599100 10.1016/j.trstmh.2008.05.011

